# The effect of shared decision-making in choosing the method of labor analgesia on childbirth experience among primiparous women

**DOI:** 10.1371/journal.pone.0274559

**Published:** 2023-02-15

**Authors:** Maryam Shahveisi, Roghaiyeh Nourizadeh, Esmat Mehrabi

**Affiliations:** 1 Department of Midwifery, Student Research Committee, Faculty of Nursing and Midwifery, Tabriz University of Medical Sciences, Tabriz, Iran; 2 Department of Midwifery, Faculty of Nursing and Midwifery, Tabriz University of Medical Sciences, Tabriz, Iran; Flinders University, AUSTRALIA

## Abstract

**Background:**

Childbearing women reported moderate and sometimes low levels of autonomy in decision-making with their health care providers especially about their pain relief type and which may affect their childbirth experience. There is limited evidence about the effect of shared decision-making about childbirth pain relief on childbirth experience and satisfaction.

**Objective:**

The present study aimed to assess the effect of shared decision-making in choosing the method of labor analgesia on childbirth experience and satisfaction among primiparous women.

**Methods:**

This interventional study was conducted on 66 primiparous women with 38–42 weeks gestational age and with symptoms of labor and childbirth onset. Women were assigned into the intervention and control groups in a ratio of 1: 1 using blocked randomization. The intervention group received shared decision making about the advantages and disadvantages of labor analgesia methods, and the control group received routine care. Questionnaires, including obstetrics and demographic characteristics, Labor Agentry Scale (LAS), McKay Childbirth Satisfaction Rating Scale (MCSRS), Support and Control In Birth (SCIB) were completed. Data were analyzed by SPSS_24_ software and independent t-test and ANCOVA were used.

**Result:**

After the intervention, the mean score of childbirth experience in the intervention group was significantly higher than that in the control group [Mean Difference (MD): 6.77, 95% CI: 2.72 to 10.82, (P <0.001)]. Further, the mean score of childbirth satisfaction in the intervention group was significantly higher than that in the control group [MD: 19.06, 95% CI: 9.63 to 28.49, (P<0.001)]. The mean score of control and support during childbirth and its subscales in the intervention group was significantly higher than that in the control group after the intervention [MD: 17.21, 95% CI: 9.40 to 25.03, (P <0.001)].

**Conclusion:**

It is recommended that mothers should be involved in treatment decisions during childbirth since they are considered an important part of providing care during labor and childbirth.

## Introduction

Based on the theory of informed and shared decision-making (SDM) about health-related issues, it has always been emphasized that informed consent-based choice is something more than just the patient’s signature on a legal document [1]. Therefore, the exchange of information between the patient and the service provider should be considered based on the patient’s values, beliefs, and preferences in decisions associated with health and treatment [2, 3].

In general, SDM is considered an essential part of patient-centered care to provide high-quality and evidence-based care [4]. The importance of participating patients in treatment decision-making in the maternity care model of the World Health Organization is evident since one of the components of care during childbirth is paying attention to the mother’s decisions. However, unfortunately, this component is ignored in most cases, especially in developing countries [5].

The experience of labor pain is a complex phenomenon, which is often experienced by a woman as the most challenging and severe pain [6] and the unfulfilled expectations of pain relief can affect women’s satisfaction with the childbirth experience [7]. Labor pain and resultant fear and anxiety make women more likely to have a cesarean section (CS) [8]. Further, they are often looking for a way to relieve labor pain if they want a normal delivery, which seems to be very effective in their highly satisfactory experience of childbirth [9].

One of the most important issues raised in SDM during labor and childbirth is the mother’s participation in choosing the type of labor analgesia. Most women are looking for a way to relieve labor pain, which seems to be very effective in their satisfactory experience of childbirth [10–12]. However, the knowledge of most women is very low about the various methods of labor pain relief and their benefits and harms in developing countries. On the other hand, some women have incorrect information and expectations regarding these methods [13]. Given that women often suffer from severe pain, anxiety, and communication barriers during childbirth, the medical staff prefers not to express the risks of analgesia in most cases when performing the analgesic procedure [6, 7, 14].

The labor care provider team should always endeavor to provide available solutions to relieve labor pain and create a pleasant and satisfying childbirth experience. Since patient labor pain relief leads to the quality improvement of treatment procedures, better cooperation of the patient with the treatment staff, more satisfaction, and ultimately, achievement of better treatment results [15].

There is limited scientific evidence regarding the effect of using shared decision-making in the care of pregnant women and their satisfaction and experience of childbirth, thus, we have arrived at some necessary conditions for SDM in maternity care. This is not a jointly sufficient condition, of course, because there are other aspects to SDM in a clinical context besides knowledge asymmetry, for example, particular extrinsic legal or policy structures, or cultural norms and there is the need for more research in this field.

Given the importance of the quality improvement of childbirth-related services through raising the level of satisfaction with childbirth experiences and considering the study gap existed in the field of SDM-based counseling regarding informed choice of labor pain relief method by parturient women and the urgent need perceived in this regard, the present study aimed to investigate the effect of SDM in choosing labor analgesia method on childbirth experience and satisfaction among primiparous women.

## Materials and methods

### Participants

This interventional study was conducted on 66 primiparous women with a low-risk pregnancy, gestational age of 38–42 weeks, and symptoms of labor referred to the prenatal clinic of the educational center of Taleghani Hospital (women’s referral hospital in northwest of Iran) in August to November 2020. The inclusion criteria included singleton pregnancy, being in the latent phase of labor (cervix dilation below 6 cm), having a low-risk pregnancy, such as the absence of bleeding during the third trimester, placental abruption, placenta previa, oligo and polyhydramnios, fetal growth disorder, and maternal chronic diseases, and having no indication for CS. The exclusion criteria were CS chosen by the mother, CS before entering the active phase (dilatation less than 6 cm), and any self-reported mental disorder. This study is part of a larger study which investigates the effect of SDM to childbirth experience, satisfaction and support and control as primary outcomes and its effect on decision regret, satisfaction and perceived shared decision making as secondary outcomes. In the present study, the childbirth experience and satisfaction were considered as the primary outcomes and the control and support during childbirth were regarded as the secondary outcomes.

### Sample size

The sample size was calculated based on the childbirth experience and satisfaction variable using G-Power software. According to the study of Madady et al. [16]. about the variable of childbirth experience and considering m_1_ = 39.0 (mean score of childbirth experience), m_2_ = 46.8 with the assumption of 20% increase in childbirth experience due to the intervention, SD_1_ = SD_2_ = 10.6, α = 0.05, and Power = 80%, a sample size of 30 was estimated for each group. According to the pilot study and regarding *m*_1_ = 19.90 (mean score of childbirth satisfaction), *m*_2_ = 23.88 with the presumption of 20% increase due to the intervention, *SD*_1_ = *SD*_2_ = 4.14, *α* = 0.05, and power = 80%, a sample size of 18 was obtained per group. Considering that the sample size calculated based on the variable of childbirth experience was higher, the final sample size regarding 10% sample loss was considered 33 per group.

### Procedures

#### Ethics statement

The sampling was started after obtaining a code of ethics from the Ethics Committee of Tabriz University of Medical Sciences (IR.TBZMED.REC.1399.184). The researcher visited the prenatal clinic of Taleghani Hospital and after introducing herself, invited all eligible women to participate in the study. Then, she briefly explained the objectives and methods of the study and those who want to participate in the study completed the written informed consent form. The pre-test questionnaire of demographic-obstetric characteristics was completed by the participants in both control and intervention groups (n = 66). The illegible women were assigned into the intervention and control groups with a ratio of 1:1 by blocked randomization using Random Allocation Software (RAS) with a block sizes of 4 and 6. The type of intervention was written on paper and placed in opaque envelopes numbered in consecutive order for allocation concealment. A non-involved person in the sampling process opened the envelopes in the order in which the participants entered the study. All of the participants in two groups were selected through simple random and one woman per day was included in the study to prevent study contamination.

### Instrumentation

#### The demographic and obstetric questionnaire

The data collected through demographic and obstetric questionnaires included age, marriage age, age of first pregnancy, age of first menstruation, educational level, occupational status, family income level, insurance status, and the number of pregnancies and childbirths.

#### The Labor Agentry Scale (LAS)

The Labor Agentry Scale (LAS), developed by Hodnett and Simmons (1987), was used to measure the childbirth experience and mothers’ feelings during childbirth. This scale with ten questions, including six positive questions and four negative questions, is scored on a 7-point Likert scale, ranging from 1 (never or almost never) to 7 (almost often). The total score range is between 10–70, as the higher the score, the more likely it is to have a positive experience [8].

#### The McKay Childbirth Satisfaction Rating Scale (MCSRS)

McKay Childbirth Satisfaction Rating Scale (MCSRS) with 40 items was used to assess childbirth satisfaction. This instrument consists of six subscales, including the satisfaction with the performance of self, midwife, partner, and physician, newborn’s status, and overall childbirth satisfaction. The responses are scored on a 5-point Likert scale, ranging from 1 (very dissatisfied) to 5 (very satisfied) and the total score range is between 34–170. The Cronbach’s alpha of 0.78 was reported for its internal consistency and the intra-class correlation coefficient (ICC) of the instrument is 0.98 [17, 18].

#### The Support and Control in Birth (SCIB)

Control and Support in birth (SCIB) scale with 33-item and three subscales, including internal control, external control, and support, was used to measure support and control during childbirth. internal control subscale with ten items assesses the control over pain, emotion, and behavior, the 11-item external control subscale focuses on pain control over decisions and procedures, and the support subscale with 12 items evaluates attitude, patience, empathy, and coping with pain. The items are rated on a 5-point Likert scale ranging from completely agree (5) to completely disagree (1). A score of 1 indicates less control and support and a score of 5 represents more control and support. The Cronbach’s alpha was reported to be between 86%–93% [4].

### Intervention

**Fig 1:**
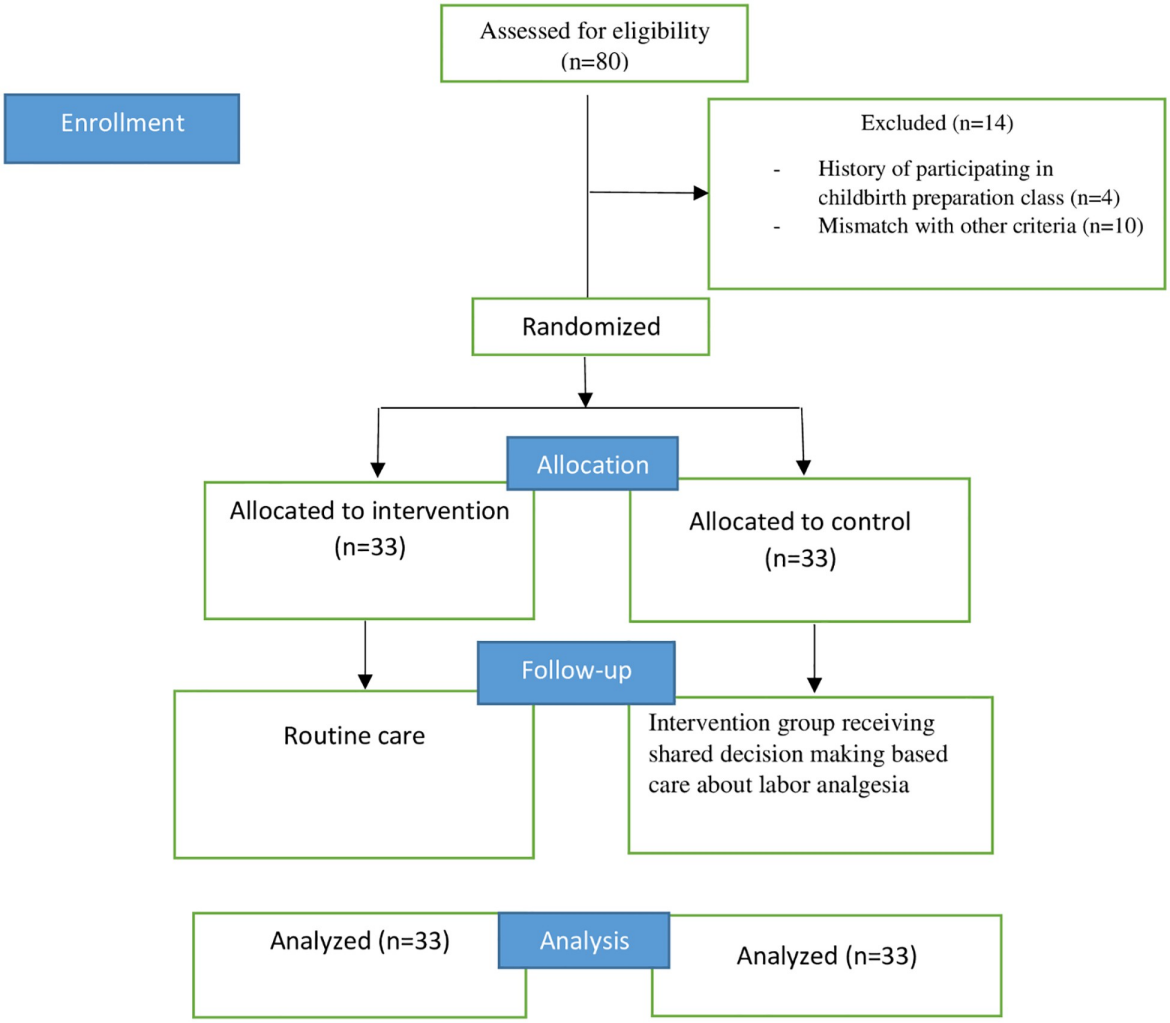
Flow chart of the study.

80 participants in the study were checked for inclusion criteria and exclusion criteria. 14 of them did not meet the inclusion criteria and were excluded from the study. 66 primiparous women with a gestational age of 38 to 42 weeks participated in the study from August to November 2020. In the follow-up and evaluation after delivery, the data of all participants in the study were analyzed. (Fig 1). In the intervention group (n = 33), the first author focused on the content of the Ottawa Standard Decision-aid Booklet about labor analgesia [19] and provided individual counseling based on SDM during latent phase of labor, when mother is stable and had not effective contractions. A complete explanation was provided about the advantages and disadvantages of using pharmacological methods of labor analgesia, including pethidine, epidural anesthesia, and remifentanil, and non-pharmacological methods of labor analgesia, including personal support, hot shower, touch and massage, aromatherapy, and Transcutaneous Electrical Nerve Stimulation (TENS). Finally, the mother participated in deciding on choosing one of the pain relief methods and control group received routine delivery care. In Iran in the routine care of vaginal childbirth, the health provider (inc. midwife or obstetrician) decide for mother and prepare brief information. All of maternal care during childbirth and their deliveries were done by the same midwife. Finally, the self-administered post-test questionnaires, including childbirth experience and satisfaction and support and control in birth were completed during the first 24 hours after delivery.

### Study outcomes

The primary outcomes of the present study included the experience and satisfaction of childbirth and the secondary outcome was support and control in childbirth, which was examined by the above tools.

### Data analysis

The SPSS_24_ software was used to analyze the data. The Kolmogorov-Smirnov test was employed to determine the normality of data distribution and independent t-test and ANCOVA were used to compare the mean scores in the two groups.

## Results

### Participants’ demographic and obstetric characteristics

The mean age of women was 23.73 (4.51) in the intervention group and 24.04 (6.81) in the control group. Most of the women (90%) in both intervention and control groups had middle school and lower education and were housewives. About 78.8% of pregnancies in the intervention group and 75.8% in the control group were wanted. Further, oxytocin was used to induce labor for 24.2% of women in the intervention group and 15.2% in the control group. In general, there was no significant difference between the two groups in terms of demographic and obstetric characteristics (Table 1). Based on the Chi-square test, the frequency of using pharmaceutical, non-pharmacological analgesia methods and no analgesia after the intervention in the intervention group decreased significantly (P <0.001), (Table 2).

**Table 1:** The socio-demographic and obstetrics characteristics of participants in the intervention and control groups.

Variable	Intervention group (n = 33) N (%)	Control group (n = 33) N (%)	P
**Age**^#^ **(year)**	23.73 (4.51)	24.06 (6.81)	0.222*
**Spouse’s age**^¥^ **(year)**	27.79 (5.04)	26.94 (6.36)	0.196*
**Level of education** ^¥^			
Secondary school	30 (90.9%)	27(81.82%)	^¥^0.213
Diploma	2(6.1%)	3(9.09%)	
University	1(3%)	3(9.09%)	
**Spouse’s education** ^¥^			
Secondary school	25(75.8%)	23(69.6%)	^¥^0.081
Diploma	5(15.2%)	6(18.18%)	
University	3(9.1%)	4(12.12%)	
**Occupation** ^¥^			
Housekeeper	30(90.9%)	26(78.8%)	^¥^0.111
Employed	3(9.1%)	7(21.2%)	
**Spouse’s occupation** ^¥^			
Worker and unemployed	21(63.6%)	14(42.4%)	^¥^0.164
Employee	3(9.1%)	4(12.1%)	
Other	9(27.3%)	15(45.5%)	
**Income level** ^¥^			
Not enough	9(27.3%)	14(42.4%)	0.426
Somewhat enough	23(69.7%)	18(54.4%)	
Enough	1(3%)	1(3%)	
**Pregnancy Type** ^¥^			
Wanted	26(78.8%)	25(75.8%)	1.000^¥^
Unwanted	7(21.2%)	8(24.2%)	
**Prescribing oxytocin** ^¥^			
No	28(84.8%)	25(75.8%)	^¥^0.537
Yes	5(24.2%)	8(15.2%)	

^#^Independent t-test

^**¥**^Chi-square test

*Mean (SD)

**Table 2:** The frequency of selected pain relief in the intervention and control groups.

Type of pain relief	Before intervention			After the intervention		
	Intervention N (%)	Control N (%)	P*	Intervention N (%)	Control N (%)	P**
**Pharmacological pain relief (remifentanil)**	26(78.7)	25(75.7)	0.736	20(60.6)	26(78.7)	>0.001
**Non-pharmacological pain relief**	2 (6.0)	3(9.09)		8 (24.4)	2 (6.0)	
**Prefer No pain relief during childbirth**	5 (15.3)	5 (15.3)		5 (15.0)	5 (15.3)	

*Chi-square test

### Childbirth experience and satisfaction

Based on ANCOVA test and adjusting the effect of confounding factor (childbirth satisfaction) and after SDM-based counseling, there was a statistically significant difference between the two groups in terms of childbirth experience. The mean score of childbirth experience in the intervention group was significantly higher than that in the control group [Adjusted Mean Difference (AMD):6.77, 95% CI: 2.72 to 10.82, (P <0.001)]. After the intervention and controlling the effect of confounding factor (control and support in birth), the mean score of childbirth satisfaction in the intervention group was significantly higher than that in the control group [AMD: 19.06, 95% CI: 9.63 to 28.49, (P <0.001)] (Table 3).

**Table 3:** The comparison of mean score of childbirth experience and satisfaction in the intervention and control groups after the intervention.

Variable	Intervention Mean (SD ^ф^)	Control Mean (SD ^ф^)	MD (95% CI**)	P
**Childbirth experience (scores range: 10–70)**				
	35.48 (4.40)	28.71 (5.39)	6.77 (2.72 to 10.82)	0.001*
**Childbirth satisfaction (scores range: 34–170)**				
	145.88 (9.35)	126.81 (11.97)	19.06 (9.63 to 28.49)	0.001*

^ф^Standard deviation

** Mean difference (MD) 95% CI

* ANCOVA test by controlling the effect of confounding factors, including childbirth satisfaction, for childbirth experience and childbirth control and support for childbirth satisfaction

### Perceived support and control during childbirth

Based on Independent T-test, after the intervention, the mean total score of support and control in birth in the intervention group was significantly higher than that in the control group [MD: 17.21, 95% CI: 9.40 to 25.03, (P<0.001)]. Further, the mean scores of the subscales of internal control, external control, and professional support in the intervention group were significantly higher than those in the control group after the intervention (P <0.001) (Table 4).

**Table 4:** The comparison of mean score of control and support during childbirth in the intervention and control groups after the intervention.

Variable	Intervention Mean (SD ^ф^)	Control Mean (SD ^ф^)	AMD (95% CI**)	P
**Total Support and control (score range: 31–155)**				
	123.5(12.53)	106.28 (10.47)	17.21 (9.40 to 25.03)	0.001*
**Subscale of internal control (score range: 10–50)**				
	34.9 (3.74)	28.5(2.73)	6.11 (3.07 to 9.18)	0.001*
**Subscale of external control (score range: 9–50)**				
	38.2 (4.08)	31.2 (4.54)	7.04 (3.92 to 10.16)	0.001*
**Subscale of professional support (score range: 9–50)**				
	29.5(4.12)	23.3 (3.76)	6.20 (2.68 to 9.76)	0.001*

*T-test

^ф^Standard deviation

**Adjusted Mean difference (MD) 95% CI

## Discussion

Based on the findings, SDM in choosing the labor analgesia improved the childbirth experience and satisfaction and support and control in childbirth. In the present study, after the intervention, the mean total score of childbirth experience in the intervention group was significantly higher than the control group. In line with the results of the present study, the results of other studies also show that supportive care has a positive role in improving the maternal experience, satisfaction, and perception of support and control during childbirth. In a qualitative study conducted by O’Hare and Fallon with the aim of determining women’s experience of control in childbirth, reported that providing supportive care and provision of emotional and psychological support on childbirth-related issues improved childbirth experience of women [20]. In another study, Citak Bilgin et al. investigated the effect of childbirth-related training on women’s perception of childbirth experience and reported that training has a positive effect on mothers’ perception of childbirth experience [21]. Accordingly, women’s lack of knowledge about childbirth-related issues causes anxiety and creates a negative childbirth experience [22]. Further, in some studies reported that women’s participation in decision-making about the childbirth process and appropriate communication with pregnant women are among the predictors of childbirth experience [23, 24]. The study results of Zhang et al. demonstrated that meeting the expectations of pregnant women by health care providers and promoting health services improves the positive childbirth experience [25]. These results are consistent with the present results and indicate the positive role of woman participation in care-related decisions during pregnancy and chilbirth; therefore, it seems that more attention should be paid to autonomy and right of mothers in order to improve their positive childbirth experience and satisfaction according of WHO (world health organization) guideline for positive childbirth experience.

Unfortunately, few interventional studies have been carried out on the effect of SDM on childbirth satisfaction and a sense of support and control. The results of some descriptive studies are presented below, which indicate factors affecting maternal satisfaction and childbirth perceived support and control. This highlights the importance of participatory decision-making-based interventions for women during pregnancy and childbirth. Consistent with the findings of the present study, in descriptive studies indicated that some factors, such as participation in decision-making, respect, and clarity during care are associated with women’s satisfaction with childbirth [26–29]. Another study presented some factors influencing childbirth satisfaction, including adapting the structure of the delivery room, applying for non-pharmacological pain reliefs, participating mothers in the childbirth process, and feeling of control over childbirth [30]. In the study of Christiaens et al. childbirth satisfaction is considered a multidimensional issue and some factors, such as the fulfillment of expectations, the ability to control oneself, and aspects related to the midwife-physician relationship with the mother are introduced as factors affecting satisfaction [31]. The above results indicate that women-centered care is an effective factor in maternal childbirth satisfaction, and since SDM is as part of women-centered care, specialists are recommended to apply SDM with emphasis on the needs and rights of mothers as well as on respecting their demands for more active involvement in childbirth, allowing them to have better control over themselves and to participate in decision-making.

The present results showed that SDM during childbirth affects maternal perceived support and control during the childbirth process. The research team finds no interventional study about the effect of SDM on labor pain management on maternal perceived support and control. The results of some related studies are presented below, which focus on childbirth support and control. In line with our study results, based on the study results of Yuenyong et al. supportive care during childbirth promotes women’s perception of support and control at birth [32]. In addition, Dugas et al. in their review study compared the effect of routine care with the care using decision aid tools during pregnancy and childbirth. The results indicated that decision support interventions increase the sense of support during pregnancy and childbirth [33]. Furthermore, the high level of care and support services during childbirth is associated with the enhancement of the feeling of support and control in birth among parturient women, increasing childbirth satisfaction [34, 35]. Colley et al. in their study entitled “women’s perception of support and control in labor and birth”, demonstrated that supporting women during childbirth through non-pharmacological pain management education and participating in decision-making during childbirth to choose the method of labor analgesia to improve the score of support and control during childbirth [36]. Additionally, Goldberg et al. in their qualitative study examined the perception of parturient women and health care providers about SDM for epidural analgesia. The findings revealed that some health care providers, especially midwives and obstetricians, often tend to express their opinions and decisions for parturient women during labor and childbirth. They reported that there is a difference between the views of physicians, delivery room health care providers, and mothers during labor and emphasized the need to adopt programs focused on improving the relationship between childbirth caregivers and parturient women for SDM [15]. Mazúchová et al in their cross-sectional study with the aim of determining the satisfaction of women with their control and participation in decision-making during childbirth which was conducted on 360 women within one year after natural birth, reported the necessity to respect the principles of women’s autonomy during childbirth, with the emphasis on providing care focused on mother’s rights as well as their active participation during childbirth, constituting an important complement to the current evidence-based approach to obstetric [37]. In general, these concluded that, a woman’s control and participation in decision-making during childbirth is an important part of maternal care. The key to global health care reform is the shift towards more patient-centered care, including their involvement in decision-making [24, 38–41], and an important aspect of care during childbirth is respect for women’s autonomy, that is, respect for their needs [10, 41].

### Strengths and limitations

Observing all the principles of interventional studies, including random allocation and allocation concealment were among the strengths of this study. In this study, standard questionnaires were used. Content design and counseling intervention were based on the cultural and moral values of the region. Also, no dropout was observed in the research participants and all those included in the study were analyzed. The limitations are that the sample size of this study was small and the follow-up period was very short and the intervention time which we preferred that counseling, using decision aid during the third trimester of pregnancy but because of our time restriction we did intervention during childbirth. In addition, it was not possible to blind the participants and data assessor due to the nature of the intervention.

## Conclusions

The results of the present study indicated the significant impact of SDM on improving the childbirth experience and satisfaction, and maternal perception of control and support during childbirth. As the provision of obstetric services and pregnancy and childbirth care by academically educated midwives has a long history in our country and given that it is considered as one of the successful countries in terms of achieving the desired level of maternal and neonatal health indicators in the Middle East, the findings of the present study can be appropriate scientific evidence for countries with moderate and poor resources. Since, the shared decision-making approach is easy, effective, and understandable for women, we recommended free-of-charge SDM services in health centers and clinics in the domain of obstetrics and gynecology, to improve the satisfaction of pregnant women. Therefore, it is recommended to conduct randomized clinical trials with larger sample size and longer follow-up periods and during pregnancy to assess the effect of shared decision-making and to draw a definitive conclusion.

## Supporting information

S1 File (SAV)
